# Preserving Pomelo Quality: Sodium Alginate Coating Containing *Bacillus subtilis* for Sustained Antifungal Activity

**DOI:** 10.3390/foods14193303

**Published:** 2025-09-24

**Authors:** Xi Wei, Yan Liu, Tingting Tang, Shanshan Lei, Jing Wu, Tianhua Ding, Xiaoyi Zhu, Weirui Fang, Jiayi Zheng, Yuxin Liu, Wen Qin, Mingrui Chen, Yaowen Liu

**Affiliations:** 1 College of Food Science, Sichuan Agricultural University, Yaan 625014, China; 2 College of Agriculture and Forestry Science and Technology, Chongqing Three Gorges Vocational College, Chongqing 404155, China

**Keywords:** pomelo, biological control, edible coating film, preservation

## Abstract

Wendan pomelo (*Citrus maxima*), valued for its unique quality and high nutritional value, is susceptible to postharvest decay caused by mechanical damage and fungal infection. This study developed a bio-based preservation strategy by incorporating *Bacillus subtilis* (*B. subtilis*) into a sodium alginate (SA)-based coating. An antagonistic *B. subtilis* strain, isolated from the pomelo growth environment, demonstrated effective inhibition against the pathogenic fungi *P. digitatum* and *P. italicum*. The *B. subtilis*/SA (2.0%) coating maintained high bacterial viability without adversely affecting the viscosity, gas barrier properties, or mechanical strength of the film. The application of the *B. subtilis*/SA coating significantly delayed fruit appearance deterioration, pulp softening, and decay in pomelo. Furthermore, the treatment enhanced flavonoid accumulation and increased the activity of antioxidant enzymes, thereby maintaining quality and extending storage life to 90 d. This study provides an effective bio-preservation strategy for the postharvest management of pomelo.

## 1. Introduction

Wendan pomelo (*Citrus maxima*) is a citrus fruit with rich nutritional and healthcare value, and its pulp is rich in nutrients such as vitamin C, flavonoids, and naringenin, which are effective in enhancing immunity, enacting antioxidant activities, and lowering blood lipids [1,2]. However, the special physiological characteristics of Wendan pomelo make postharvest preservation a great challenge: the fragile skin of the fruit is susceptible to microbial infestation due to mechanical damage during postharvest distribution, resulting in an increased decay rate; the vigorous respiratory metabolism of Wendan pomelo after harvest is accompanied by significant water loss and quality degradation, which greatly restricts the shelf-life and negatively affects the sales of the fresh fruits [3,4]. Therefore, it is important to develop preservation technologies that can prolong the storage period of Wendan pomelo and maintain good eating quality for the healthy development of the pomelo industry.

In recent years, research on sustainable postharvest disease control strategies has expanded to include diverse microbial resources and the valorization of agricultural byproducts. Volentini et al. [5] demonstrated that cell-free supernatants (CFSs) isolated from fermented foods effectively inhibited green and blue molds on lemons, primarily due to the production of organic acids and phenolic compounds. Furthermore, epiphytic microorganisms (e.g., yeasts and bacteria) from citrus fruit surfaces have been proven to serve as effective biocontrol agents by competing for nutrients and space or secreting antimicrobial compounds to suppress pathogen growth [6]. On the other hand, circular economy strategies encourage the conversion of agricultural processing byproducts (e.g., fruit peel) into high-value products. Riolo et al. [7] developed a bioactive formulation based on lemon peel powder fermented by LAB, which not only effectively controlled blue mold on citrus fruit but also embodied a green and sustainable postharvest management philosophy.

Antagonistic bacteria can not only produce bacteriostatic substances but also act as bioactivators to induce plant disease resistance [8]. Terao et al. [9] found that the antagonistic yeast CMAA-1112 reduced the incidence of the green mold in orange fruit by activating the immune defense system. However, the direct application of antagonistic bacteria makes the antimicrobial preservation performance of antagonistic bacteria time-sensitive due to the limited nutrients on the surface of the fruit and the unstable survival environment [10,11]. In order to solve these problems, the antagonistic bacteria can be embedded in the edible coating using encapsulation technology to maintain the higher activity and application range of antagonistic bacteria [12]. It is noteworthy that *Bacillus subtilis* (*B. subtilis)*, as an important biocontrol agent, has also shown potential in controlling postharvest diseases of citrus. For example, *B. subtilis* B-912 was proven to significantly inhibit green and blue mold in citrus fruits [13]. However, existing research has mostly focused on the direct application of bacterial cells or fermentation liquids; meanwhile, integrated approaches, combining antagonistic bacteria (e.g., *B. subtilis)* with edible coatings to achieve the synergistic effects of physical protection, microenvironment stabilization, and biocontrol, remain relatively unexplored.

Coating is the predominant preservation method used for fruits and vegetables. These coatings regulate gas exchange across production surfaces while providing physical protection against mechanical damage [14,15,16]. Sodium alginate (SA), as a natural anionic polysaccharide polymer extracted from marine algal resources, can be used to prepare gel and film materials for food preservation due to its weak gelling property, viscosity, and film-forming property [17]. Studies have shown that SA and ZnO coatings can extend the shelf life of oranges (*Citrus sinensis* L.) to 20 d [18]. Chiumarelli et al. [19] and Robles-Sanchez et al. [20] demonstrated that an SA coating combined with antioxidant compounds (i.e., citric acid and ascorbic acid) could maintain the quality and nutritional value of fresh mangoes when stored at 4 °C for 12 d.

Antagonistic bacteria are small in size and do not damage the original characteristics of the coating [21], and the bacteriostatic substances produced by the antagonistic bacteria can diffuse to the surrounding area through the gap in the coating to achieve bacteriostatic preservation [22]. Torres-García et al. (2024) [23] added *B. subtilis* into the coating for strawberry preservation. The results showed that the coated film containing *B. subtilis* could effectively inhibit the growth of molds on the surface of strawberries and prolong the freshness period of strawberries to maintain better eating quality. The coating can also provide stable living conditions for antagonistic bacteria so that they can maintain high activity for a longer period [24,25]. This is a safe and non-polluting new strategy for disease control that stimulates the intrinsic disease resistance mechanism of fruits through biotic or abiotic excitation factors to achieve postharvest disease control. It has been verified that *Bacillus sphaericus* and chitosan (CS), as activating factors, can induce the enhancement of fruit resistance by promoting the expression of resistance-related genes in the secondary metabolic pathways of fruits, as well as affecting the biosynthesis of secondary metabolites and the accumulation of resistance-related metabolites in antioxidant-related metabolic pathways [26,27,28].

This study developed a novel biocontrol system by integrating antagonistic microbes with edible coatings for the preservation of Wendan pomelo. The aims of this study were: (i) to screen antagonistic bacteria with significant inhibitory effects against postharvest pathogens of Wendan pomelo and identify a suitable coating substrate; (ii) to ensure the survival rate and activity stability of the antagonistic bacteria within the coating system through process optimization; and (iii) to evaluate the efficacy of this biocontrol system in prolonging the storage period of Wendan pomelo by inducing disease resistance and maintaining postharvest quality stability.

## 2. Materials and Methods

### 2.1. Materials

SA (food-grade) was provided by Lianyungang Tiantian Seaweed Industry Co., Ltd. (Lianyungang, China); CS (food-grade) was purchased from Zhengzhou Mingze Biotechnology Co., Ltd. (Zhengzhou, China); Konjac glucomannan (KGM, food-grade) was obtained from Zhejiang Tianhe Food Biotechnology Co., Ltd. (Lishui, China); Sodium carboxymethyl cellulose (CMC-Na, food-grade) was provided by Shanghai Changguang Enterprise Development Co., Ltd. (Shanghai, China). *Penicillium italicum* (*P. italicum*) and *Penicillium digitatum* (*P. digitatum*) were provided by the Key Laboratory Department of Agro-Products Processing and Storage, Sichuan Agricultural University (Yaan, China). All other chemicals and reagents were of analytical grade and purchased from Chengdu Cologne Chemical Reagent Company (Chengdu, China).

The Wendan pomelos used in this study were harvested from orchards in Guang’an city, Sichuan Province, China. A number of healthy leaves and fruits of Wendan pomelo were picked and placed in self-sealing bags, while fruits with obvious symptoms of disease were also collected and placed into other self-sealing bags and numbered; the sampling locations, time, and other data were recorded in detail. After sampling, the samples were immediately transported to the laboratory and stored in a refrigerator (4 °C) to maintain their biological activity. The antagonistic bacteria isolated from Wendan pomelo were identified as *B. subtilis*. The identification results are shown in Figure S1.

The sequencing results of the antagonistic bacterial strain were aligned using the online BLAST tool provided by the National Center for Biotechnology Information (NCBI) (https://blast.ncbi.nlm.nih.gov/Blast.cgi, accessed on 17 September 2025). Based on the homologous sequence results, the related sequences with higher similarity were selected for alignment and analysis. The phylogenetic tree was constructed using the Tamura3-parameter model and the Maximum Likelihood method. The antagonistic bacterial strain is in the same branch as *B. subtilis* NBRC13719, and the nucleotide sequence similarity is as high as 100%. The results indicate that they have the closest genetic relationship. Based on morphological characteristics and molecular biology, the antagonistic bacteria strain was identified as *B. subitlis*. The 16S rRNA gene sequence of this strain has been preserved in the GenBank database, with the registration number NR_112629.1.

### 2.2. Assessment of B. subtilis Antagonistic Capacity

#### 2.2.1. Determination of the Inhibition Spectrum

A pathogenic fungal cake (5 mm in diameter) was accessed at the center of the potato dextrose agar (PDA) plate, and a *B. subtilis* cake of the same specification was inoculated 2 cm from the center; the control group was set as a blank PDA. Then, 7 d of incubation at 28 °C was followed by the determination of the diameter of the pathogenic fungal colonies. The antagonistic inhibition rate (R) was calculated by the following formula:

(1) $$R\left(\%\right)=\frac{R_{1}-R_{2}}{R_{1}}\times 100$$

where R is the inhibition rate, R_1_ is the colony diameter of the pathogenic fungi in the control group, and R_2_ is the colony diameter of the pathogenic fungi in the treatment group.

#### 2.2.2. Determination of Inhibitory Capacity of Volatile Metabolites of *B. subtilis* Against Pathogenic Fungi

A measure of 100 μL of *B. subtilis* bacterial suspension (1 × 10^8^ cfu/mL) was aspirated and coated to prepare the bacteria-containing plate. Subsequently, the pathogenic fungal cake (5 mm in diameter) was accessed in the center of the PDA plate, which was docked with the bacteria-containing plate. Blank lysogeny broth (LB) plates were set as the blank controls and incubated at 28 °C for 7 d. The diameters of the pathogenic fungal colonies were measured. The formula for calculating the inhibition rate was as follows:

(2) $$\mathrm{Inhibition}\;\mathrm{rate}\;\left(\%\right)=\frac{\mathrm{control}\;\mathrm{colony}\;\mathrm{diameter}-\mathrm{treatment}\;\mathrm{colony}\;\mathrm{diameter}}{\mathrm{control}\;\mathrm{colony}\;\mathrm{diameter}-\mathrm{colony}\;\mathrm{diameter}}$$

#### 2.2.3. Spatial and Nutrient Competition in *B. subtilis*

The, we aspirated 200 μL of the spore suspension to coat the plate as the control group. In the experimental group, 100 μL of *B. subtilis* suspension was mixed with 100 μL of the spore suspension of the pathogen and incubated at 28 °C for 2–3 d to observe the results.

#### 2.2.4. Effects of *B. subtilis* on Morphology and Spore Germination of *P. digitatum* and *P. italicum*

The spore suspensions of the two pathogenic fungi were first prepared, and the concentration was diluted to 10^5^ cfu/mL with a PDB gradient; then, 20 μL was pipetted and added to a pre-sterilized single-concave slide recess. An equal volume of *B. subtilis* suspension (10^8^ cfu/mL) was added to the experimental group, and the control group was replaced with an equal amount of sterile water. After covering the coverslips, they were transferred to Petri dishes (9 cm) lined with moist filter paper at the bottom and incubated at 28 °C for 24 h. Spore germination was observed under the microscope at 10 h and 24 h, respectively. The germination was determined by the length of the germ tube exceeding the length of the spore itself, and no less than 200 spores were counted under the same field of view for each observation.

### 2.3. Screening of Coating Substrates

Different masses of SA (0.5, 1.0, 1.5, 2, and 2.5 g) and 2.0 g of glycerol were added to 100 mL of distilled water and stirred well to form the SA coating; different masses of CS (0.5, 1.0, 1.5, 2.0, and 2.5 g) were then dissolved in 1% sterile acetic acid, supplemented with 1% glycerol, and adjusted to 100 mL with 1% acetic acid. Dissolution was completed at 40 °C with stirring (1 h), which was followed by sonication (40 kHz, 15 min); KGM powder (0.5, 1.0, 1.5, 2.0, and 2.5 g) was hydrated in 300 mL distilled water at 58 °C (40 min stirring), and then sonicated (20 min) to remove bubbles; CMC-Na (0.5, 1.0, 1.5, 2.0, and 2.5 g) was dispersed in 100 mL distilled water and stirred (800 rpm, 25 °C, 2 h) until complete dissolution was achieved.

Four coating solutions were mixed with a *B. subtilis* suspension (1 × 10^8^ cfu/mL) in a 9:1 volume ratio. Under the condition of storage at room temperature, 1 mL of the mixed sample solution was removed every 10 d, inoculated on LB plates after gradient dilution, and counted after 48 h of incubation at 37 °C. The biocompatibility of each coating substrate was evaluated by comparing the viable bacterial counts on different substrates, and the most suitable coating substrate was screened.

### 2.4. Effect of Different Coatings on the Morphology of B. subtilis

Colonies of *B. subtilis* from biocompatibility assays were streaked onto fresh LB agar plates and incubated at 37 °C for 48 h to obtain isolated colonies. Selected colonies were inoculated into LB liquid medium and incubated at 37 °C with shaking (120 rpm) for 24 h, followed by the formation of a bacterial film on the liquid surface.

### 2.5. Determination of Optimal In Vitro Inhibitory Concentration

After gradient dilution of *B. subtilis* suspension, 100 μL of the dilution was pipetted and spread evenly on the surface of PDA plate, and the blank CK group was added with equal amount of sterile water. A hole was punched in the center of the plate using a sterile perforator and a pre-prepared pathogen cake (5 mm in diameter) was inoculated into the hole. The plate was incubated at 28 °C for 4–5 d, and the diameter of the colonies was measured by the crossover method.

### 2.6. Determination of Optimal In Vivo Inhibitory Concentration

Two wounds (5 mm diameter × 4 mm depth) were created on opposite sides of the pomelo peels. A measure of 10 μL of the *B. subtilis* suspensions (1 × 10^8^ cfu/mL) was inoculated into the fruit wounds and air-dried, followed by inoculation with 10 μL *P. italicum* spore suspension (1 × 10^5^ cfu/mL). After sealing the wound with plastic wrap, the fruits were incubated in a humidity-controlled incubator (T: 15 ± 1 °C, RT: 75–85%). The CK group was inoculated with an equal volume of sterile water instead of a bacterial suspension.

### 2.7. Characterization of Coating Properties

#### 2.7.1. Water Vapor Permeability (WVP)

Determination of water vapor transmission rate by the method of Luo et al. [29].

#### 2.7.2. Determination of Viscosity

The viscosities of the SA coatings at different concentrations were determined using an MCR 102e rheometer (Anton Paar, Graz, Austria), equipped with a parallel-plate geometry (50 mm, 1 mm gap). The apparent viscosity of SA coating was measured at 25 °C.

#### 2.7.3. Mechanical Performance Tests

The samples were cut into strips measuring 15 mm × 120 mm. The tensile strength (TS) and elongation at break of the films were evaluated using a universal testing machine equipped with a load cell of 500-N (4500, Instron Corporation, Canton, MA, USA) at a tensile rate of 50 mm/min. The average values of five measured samples were reported. The TS and EB values of the films were calculated using the following equations:

(3) $$\mathrm{TS}\;=\frac{F}{A}$$

(4) $$\mathrm{EB}=\frac{L-L_{0}}{L_{0}}\times 100$$

where TS is measured in MPa, F is the maximum tension (N) when the film breaks, A is the cross-sectional area (mm^2^) of the film before the test, L is the length of the film before the test (mm), and L_0_ is the length of the film at the test break (mm).

#### 2.7.4. Scanning Electron Microscope (SEM)

The epidermis was cut on the surface of Wendan pomelo in the size of 1 cm^2^, quick-frozen in liquid nitrogen, vacuum freeze-dried, sprayed with gold, and observed by electron microscopy.

### 2.8. Determination of In Vivo Antibacterial Properties

Three equidistant wounds (4 mm diameter × 5 mm depth) were symmetrically prepared in the equatorial region of the pomelo using a sterile perforator. Each wound received 30 μL of either 2.0% SA coating, *B. subtilis*/SA composite coating, or sterile water (CK group). After 10 min, 30 μL of *P. italicum* spore suspension (5 × 10^4^ CFU/mL) was added. Following a second 10 min incubation, wounds were sealed with cling film, and fruits were stored at 25 ± 1 °C with 85–95% relative humidity.

### 2.9. Determination of B. subtilis Viability

*B. subtilis* viability was determined by the method of Fernandes et al. [30].

### 2.10. Coating Samples Preparation

Uniform-sized Wendan pomelos with undamaged peels were selected. The fruits were immersed for 30 s in either 2.0% SA coating solution, *B. subtilis* suspension (10^8^ cfu/mL), *B. subtilis*/SA composite coating, or sterile water (CK group), with 25 fruits per treatment group. After treatment, all fruits were stored at 8 ± 1 °C with a relative humidity of 75–85% for 90 d. Each test consists of three biological replicates.

### 2.11. Determination of Quality Indexes of Wendan Pomelo

#### 2.11.1. Appearance and Flesh Color

Wendan pomelos were randomly selected. Images of the peel and flesh sections of pomelo were captured using a SONY IMX989 camera (Sony Corporation, Tokyo, Japan). The color parameters of the peel were then measured quantitatively using a precision colorimeter (Hangzhou Spectral Technology Co., Ltd., Hangzhou, China). Each test consists of three biological replicates.

#### 2.11.2. Hardness, Weight Loss Rate, Total Suspended Solids (TSS) and Total Acid (TA)

The weight loss rate, TA content, and TSS were determined as described by Xie et al. [31]. A hand-held GY-4 fruit hardness tester with a 2 mm diameter cylindrical probe (Yueqing Edbao Instrument Co., Ltd., Yueqing, China) was used to measure the hardness of each pomelo (the skin was cut off to reveal the flesh). Each test consists of three biological replicates.

### 2.12. Investigation of Physiological Mechanisms of B. subtilis/SA Coatings in the Postharvest Preservation of Pomelos

In the equatorial region of the pomelo, three equidistant wounds (4 × 5 mm) were symmetrically prepared using a sterile perforator. Each wound was injected with 20 μL of *B. subtilis*/SA composite coating, while controls received an equal volume of sterile water. After air-drying at room temperature until complete solvent evaporation, the wounds were sealed with a food-grade clinging film. Fruits were then transferred to a climate-controlled incubation system (20 ± 1 °C, 90–95% RH) for incubation.

### 2.13. Flavonoids and Total Phenolics Content

The flavonoids and total phenols in the pulp of the pomelo were determined using the method described by Zhang et al. [32]. Each test consists of three biological replicates.

### 2.14. Determination of Resistance-Related Enzyme Activities

The enzyme activities of the fruit tissue samples were measured using specific ELISA kits (Jiangsu Jingmei Biotechnology Co., Ltd., Yancheng, China) for peroxidase (POD), polyphenol oxidase (PPO), ascorbate peroxidase (APX), phenylalanine ammonia-lyase (PAL), catalase (CAT), and superoxide dismutase (SOD). Each test consists of three biological replicates.

### 2.15. Statistical Analysis

All the experiments were set up with at least 3 biological replicates. The results were expressed as mean ± standard deviation (SD). Analysis of variance (ANOVA) was applied to determine the significant differences between means (*p* < 0.05). Post hoc analysis was conducted using the Tukey’s honest significant difference (HSD) test with a significance level set at *p* < 0.05. Graphics were drawn using Origin 2021.

## 3. Results and Discussion

### 3.1. Antagonistic Capacity of B. subtilis

#### 3.1.1. Antifungal Spectrum of *B. subtilis*

**Figure 1:**
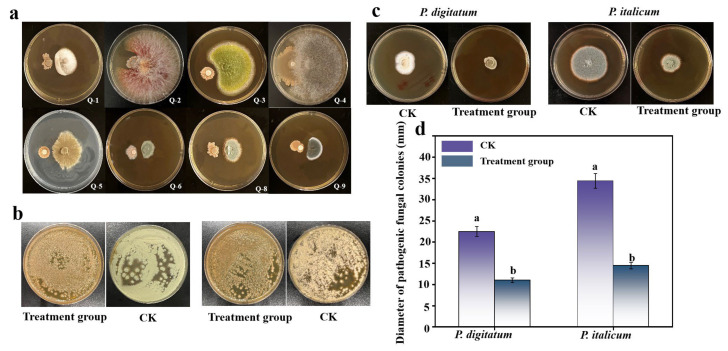
The effect of *B. subtilis* on the growth of fungal colonies (**a**); determination of in vitro hydrolytic enzyme activity of *B. subtilis*, and spatial and nutritional competition between *B. subtilis* and pathogenic fungi (**b**); determination of volatile antibacterial activity of *B. subtilis* (**c**,**d**). The data are shown as mean ± S.E. (n = 3) and different letters (a,b) represent the significant differences (*p*  <  0.05).

The results of the plate confrontation are shown in Figure 1a. The antagonistic strains had different degrees of inhibitory effects on the fungi sieved from the surface of Wendan pomelo, among which *P. italicum* and *P. digitum* had the best inhibitory effect, and were able to inhibit the growth of mycelium effectively.

#### 3.1.2. Nutritional and Spatial Competition

As shown in Figure 1b, in the plates coated with *P. italicum* and *P. digitum* spore suspensions after the addition of *B. subtilis* suspensions, there was almost no growth, and the antagonist fungus dominated the growth; in the plates without the addition of *B. subtilis* suspensions, the mycelium of the pathogenic fungus grew all over the plates and produced a large number of spores. The results surface that *B. subtilis* can inhibit the growth of pathogenic fungi by competing with them for nutrients and space.

#### 3.1.3. Assessment of Volatile Antimicrobial Activity in *B. subtilis*

*B. subtilis* also demonstrated antifungal activity through volatile compound production. Figure 1c,d reveal that volatiles emitted by *B. subtilis* inhibited mycelial growth of *P. italicum* and *P. digitatum* by 42.3% and 49.2%, respectively. Treated fungal colonies showed significantly reduced diameters compared to untreated controls. Furthermore, the treatment group exhibited sparser mycelial density, thinner colony morphology, and diminished spore production relative to controls. These findings demonstrate that volatile metabolites from *B. subtilis* effectively suppress hyphal expansion and decelerate pathogenic fungal proliferation.

#### 3.1.4. Effects of *B. subtilis* on Mycelium and Spores of Pathogenic Fungi

**Figure 2:**
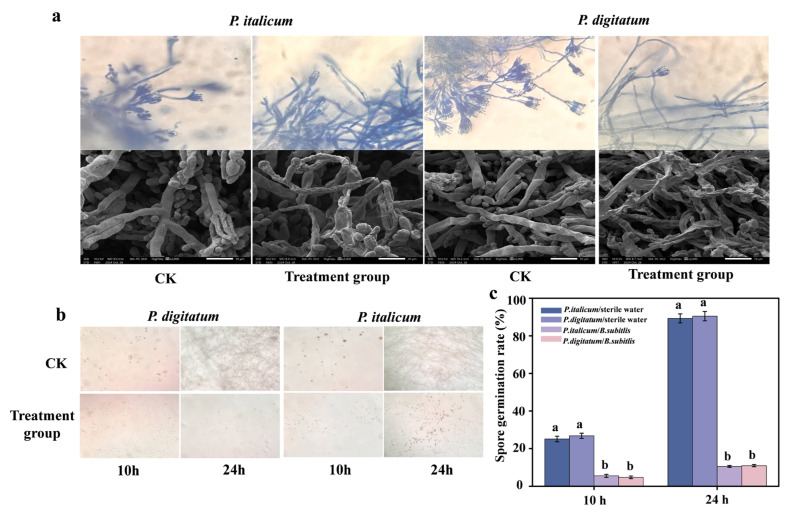
Optical microscopy and SEM images of pathogenic fungal hyphae and spores treated with antagonistic bacteria (**a**). The effect of *B. subtilis* suspension on the growth of pathogenic fungal hyphae (**b**) and spore germination of pathogenic bacteria (**c**). The data are shown as mean ± S.E. (n = 3) and different letters (a,b) represent the significant differences (*p * <  0.05).

Optical microscopy showed untreated hyphae with uniform staining, smooth surfaces, abundant conidiophores, and dense spore clusters (Figure 2a), contrasting with the *B. subtilis*-treated hyphae near inhibition zones; these exhibited irregular staining, fragmentation, translucency, and reduced conidiophores and spore numbers. The SEM results revealed significant structural alterations in the treated samples: they contracted hyphae with roughened surfaces and spores displaying severe deformation, pitting, and aggregation. Spore counts were markedly lower in the treated groups than in the control groups. Untreated controls consistently showed uniform, linear tubular hyphae with smooth surfaces and morphologically intact, smooth spores. These results indicate that *B. subtilis* suppresses fungal growth by physically damaging hyphal integrity and spore morphology.

The mycelial growth of different treatments after 10 h and 24 h of incubation was observed using light microscope as shown in Figure 2b, and the *B. subtilis* suspension treatment significantly inhibited the germination of *P. italicum* and *P. digitum* spores as compared to the blank control group.

Figure 2c illustrates *B. subtilis* inhibition of spore germination in *P. italicum* and *P. digitatum*. Control germination rates were 45.09% (10 h) and 89.45% (24 h) for *P. italicum* versus 5.63% and 8.18% in treated groups, and 28.65% (10 h) and 84.42% (24 h) for *P. digitatum* controls versus 5.08% and 10.94% with treatment. Statistical analysis confirmed significant inhibition rates of 87.5% (*P. italicum*) and 82.2% (*P. digitatum*) at 10 h, and 90.8% and 87.0% at 24 h (*p* < 0.05).

### 3.2. Biocompatibility of Different Coating Substrates

**Figure 3:**
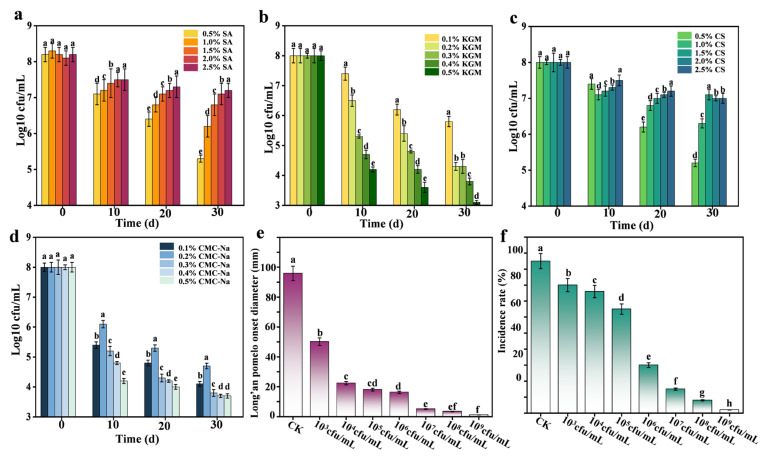
SA (**a**), KGM (**b**), CS (**c**), and CMC-Na (**d**). Determination of antagonistic bacterial activity in different coating matrices. Effect of *B. subtilis* suspension with different concentrations on fruit disease diameter (**e**) and fruit incidence rate (**f**). The data are shown as mean ± S.E. (n = 3) and different letters (a–h) represent the significant differences (*p*  <  0.05).

To identify the optimal coating substrate for encapsulating *B. subtilis*, we evaluated the activity using various concentrations of different substrate materials. As shown in Figure 3a, *B. subtilis* maintained high activity on coatings made of SA. Activity increased with increasing SA concentration until a decline occurred at 2.0% SA. This decrease may have stemmed from the denser gel structure formed at higher SA concentrations, which likely restricted oxygen diffusion and creates hypoxic conditions that impaired microbial metabolism and reduced *B. subtilis* viability within the coating [17]. The KGM-based coatings (Figure 3b) and *B. subtilis* showed consistent low activity. This was attributed to abundant hydroxyl groups of KGM, which readily form hydrogen bonds with water. Even at low concentrations, KGM rapidly formed colloids, and higher concentrations exacerbated this behavior. The resulting uneven distribution of *B. subtilis* within the viscous matrix leads to unstable *B. subtilis* activity. CS-based coatings (Figure 3c) supported high *B. subtilis* activity, with the activity increasing proportionally with CS concentration. In the CMC-Na coatings (Figure 3d), *B. subtilis* activity remained low, likely due to the high viscosity and osmotic pressure of the CMC-Na solutions. Elevated concentrations induced osmotic stress, causing cellular dehydration and impairing *B. subtilis* viability [33]. In summary, the activity of *B. subtilis* in the coatings prepared using SA and CS was stable and good.

### 3.3. Antagonistic Bacterial Morphology

The morphological effects of the coatings on *B. subtilis* are shown in Figure S2a. Uncoated *B. subtilis* (CK group) formed yellowish-white opaque colonies on LB agar plates with wrinkled surfaces and irregular edges. In liquid culture, it produced a smooth, dense, white biofilm on the medium surface (Figure S2(a_1_)). When encapsulated in CS coatings, *B. subtilis* colonies on LB plates were smaller and more opaque, and they displayed smoother edges than those of the control. The film formed on the surface of the liquid medium was discontinuous and not as smooth as that of the CK group (Figure S2(a_2_)). In the SA coatings, colonies retained a yellowish-white, opaque appearance with wrinkled centers and uneven edges on LB plates, and the colonies formed on the surface of the liquid medium had the same morphology as those of the CK group (Figure S2(a_3_)). *B. subtilis* inoculated with KGM and CMC-Na did not show any significant change in the morphology of the colonies formed on the plate, but the colony film formed in the liquid medium was the most different from that of the CK group and could not form a complete and continuous colony film on the surface of the liquid (Figure S2(a_4,_a_5_)). These results indicated that SA coatings minimally altered *B. subtilis* morphology, whereas CS caused minor structural changes. In contrast, KGM and CMC-Na significantly disrupted biofilm integrity. As bacterial morphology correlates with antagonistic efficacy, SA was selected as the optimal substrate for encapsulating *B. subtilis*.

### 3.4. Screening of Optimal Inhibitory Concentrations of B. subtilis

Figure S2b showed that *P. italicum* colony diameter decreased progressively with increasing *B. subtilis* suspension concentration. Colonies exposed to 1 × 10^8^ or 1 × 10^9^ cfu/mL exhibited the smallest diameters and >90% inhibition, significantly lower than at 1 × 10^6^ or 1 × 10^7^ cfu/mL. These results indicate optimal in vitro inhibition of *P. italicum* at 1 × 10^8^ and 1 × 10^9^ cfu/mL, with no significant differences between them. Consequently, 1 × 10^8^ cfu/mL was selected for subsequent experiments.

### 3.5. Optimal Inhibitory Concentrations In Vivo

Figure 3e,f and Figure S2c show the effects of *B. subtilis* concentration on pomelo disease incidence and lesion diameter. Disease incidence (Figure 3f) decreased progressively with concentration, dropping below 2% at 1 × 10^8^ and 1 × 10^9^ cfu/mL, a statistically significant reduction compared to 1 × 10^7^ cfu/mL. These results indicate optimal in vivo inhibition of *P. italicum* at 1 × 10^8^ or 1 × 10^9^ cfu/mL, with no significant difference between them. Consequently, 1 × 10^8^ cfu/mL was selected as the optimal in vivo concentration.

### 3.6. Characterization of SA Coating Films at Varying Concentrations

#### 3.6.1. WVP

**Figure 4:**
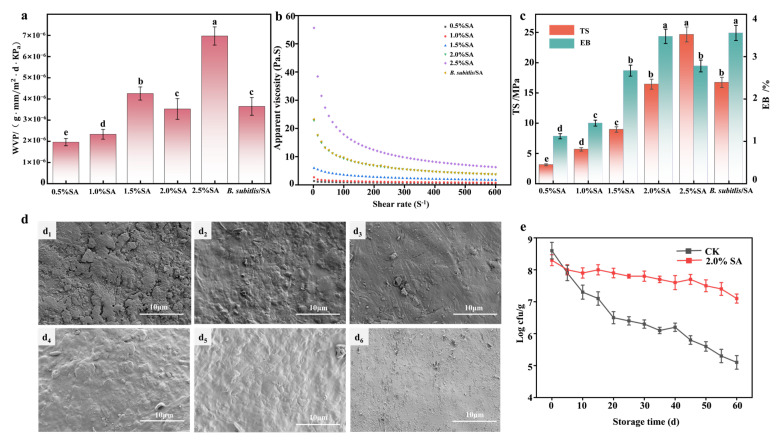
The effect of SA concentration on the barrier properties of membranes (**a**), TS and elongation at break of membranes (**b**), and membrane viscosity (**c**). Different concentrations of SA coating 0.5% (**d_1_**), 1.0% (**d_2_**), 1.5% (**d_3_**), 2.0% (**d_4_**), 2.5% (**d_5_**), and *B. subtilis*/SA (**d_6_**) SEM image of *B. subtilis*/SA coating on fruit peel; *B. subtilis* activity in coatings (**e**). The data are shown as mean ± S.E. (n = 3) and different letters (a–e) represent the significant differences (*p*  <  0.05).

WVP is inversely related to moisture barrier efficacy, crucially preserving food freshness by reducing vapor transmission [34]. Figure 4a showed a nonlinear WVP response of SA films to increase SA concentration: WVP peaked at 1.5%, declined until 2.0%, and subsequently rose. The WVP value at 2.0% SA concentration was significantly lower than that in the 1.5% and 2.5% concentration groups (*p* < 0.05). The decline (1.5–2.0%) likely resulted from reduced intermolecular gaps, forming a denser, less permeable structure. However, at 2.5% SA, excessive SA caused molecular aggregation, increasing film rigidity, brittleness, and WVP. Crucially, lower WVP reduced moisture evaporation in Wendan pomelos, slowing storage weight loss. Therefore, 2.0% SA was optimal for coating formulation. Notably, *B. subtilis* incorporation did not significantly alter the WVP of the coating (*p* > 0.05).

#### 3.6.2. Viscosity

Figure 4b shows the shear-thinning behavior observed in the SA coating solutions, with viscosity decreasing as shear rate increased. This non-Newtonian response likely stems from disruption of hydrogen-bonded network under shear stress, reducing intermolecular interactions. At 2.5% SA, excessive viscosity impaired the flow uniformity and increased fracture risk during application. Conversely, concentrations < 2.0% resulted in low viscosity, causing poor adhesion and incomplete film formation. The 2.0% SA solution exhibited optimal viscosity for complete coating deposition [35]. However, the incorporation of *B. subtilis* did not significantly affect the viscosity of the 2.0% SA solution.

#### 3.6.3. Mechanical Strength

Figure 4c shows the tensile strength and elongation at break of SA films at varying concentrations. The TS increased with increasing SA concentration, reaching a maximum of 2.0%. In contrast, the elongation at the break decreased progressively at higher SA concentrations. This inverse trend was attributed to the enhanced rigidity in denser SA matrices, which increased the brittleness of the coating and compromised the coating’s integrity [36]. The incorporation of *B. subtilis* into the 2.0% SA film did not significantly affect the mechanical strength of the coating film.

#### 3.6.4. SEM

Figure 4d presents SEM images of pomelo surfaces with varying SA coating concentrations. Untreated fruit (Figure 4(d_1_)) showed discontinuous native wax patches. The 0.5% SA coating (Figure 4(d_2_)) unevenly covered the surface with visible underlying wax. Uniformity improved at 1.0% SA (Figure 4(d_3_)), though residual wax remained visible. Both 2.0% (Figure 4(d_4_)) and 2.5% SA (Figure 4(d_5_)) completely covered epidermal waxes, with the 2.0% coating exhibiting superior flatness. The *B. subtilis*/SA composite coating (Figure 4(d_6_)) showed the enhancement of continuity and flatness compared to 2.0% SA alone, with uniform micro-protrusions indicating homogeneous bacterial dispersion.

### 3.7. In Vivo Bacteriostatic Effect of B. subtilis/SA Coating Solution

Wendan pomelo peels are prone to mechanical damage during postharvest storage, and pathogenic fungi can infect the fruit through wounds, causing disease. As shown in Figure S3, after inoculation of Wendan pomelo wounds with pathogenic fungal spores, the control group showed browning and rotting at 3 d, and the rotting area expanded at 4 d. Pathogenic fungi were growing in the wounds, and the lesion diameter in the control group reached 9.46 ± 0.46 cm at 5 d (Table 1), with vigorous fungal growth and abundant spore production. In contrast, Wendan pomelo treated with 2.0% SA coating showed initial pathogenic fungal growth at 5 d but exhibited no significant browning or rotting. Pomelo treated with *B. subtilis*/SA coating displayed no pathogenic fungal growth, browning, or rotting at 5 d. These results indicate that the *B. subtilis*/SA coating can inhibit pathogenic fungi for extended periods, providing effective antimicrobial and freshness-preserving effects.

### 3.8. B. subtilis Viability in Coating

As shown in Figure 4e, *B. subtilis* viability within the SA coating remained consistently high (10^6^ cfu/g) during 60 d of storage, and the viability did not show significant fluctuation during the entire storage process. In contrast, the CK group exhibited a progressive decline in bacterial viability, dropping below 10^7^ cfu/g by 20 d and further decreasing to 10^5^ cfu/g by 60 d. The results demonstrated that *B. subtilis* was able to maintain high activity in the coating, which may be due to the SA coating providing a stable environment and nutrients for bacterial survival. This finding is consistent with those presented by Fernandes, de Oliveira, and de Souza [30].

### 3.9. Effect of Different Treatments on the Quality of the Pomelo

#### 3.9.1. Appearance and Color

**Figure 5:**
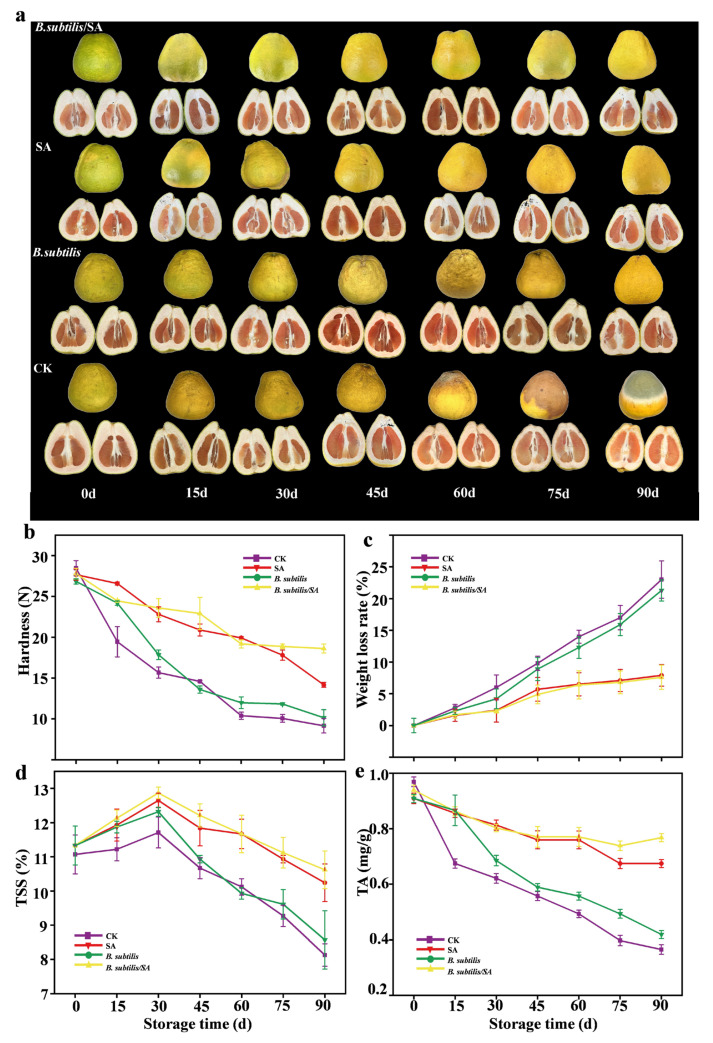
Changes in the appearance (**a**), hardness (**b**), weight loss (**c**), TSS (**d**), and TA (**e**) of the pomelo during storage. Detailed data and statistics are provided in the Supplementary Materials, Table S1.

Figure 5a illustrated the appearance changes in the pomelo during storage. Initially, all the groups exhibited yellow–green peels. By 30 d, the CK group showed complete yellowing, whereas the 2.0% SA and *B. subtilis*/SA composite coating groups retained a stable yellow–green coloration. The cross-section revealed that the firmness, color, and water content of the flesh in treated groups remained acceptable at 30 d. The CK group developed browning and hollowing of the peel at 60 d (no rebound when pressed), and moldy spots appeared on the fruit stalks at 75 d. The composite-coated and SA groups maintained their complete structure at 90 d, and the indicators of the flesh tissue were significantly better than those of the CK. The *B. subtilis*/SA composite coating effectively delayed visual deterioration during storage, demonstrating superior preservation efficacy compared to the individual treatments.

Color difference results (Figure S4) showed that, from 0 d to 90 d, the b* value increased in the CK group (36.57 to 45.89) but remained relatively stable in the *B. subtilis*/SA coating group (40.85 to 42.99). Concurrently, the L* value decreased sharply in the control (52.34 to 46.73) compared to a minimal decrease in the *B. subtilis*/SA group (53.24 to 52.89). The *B. subtilis*/SA coating effectively inhibited the b* increase and L* decrease in pomelo peel, likely by regulating the fruit microenvironment, acting as a gas/humidity barrier, reducing respiration and nutrient loss, and slowing enzymatic browning. The L* decrease and b* of the CK group increase might result from brown pigment formation and chlorophyll degradation during aging [37]. The sustained high L* value in the coated group indicates its protective role on Wendan pomelo color during storage.

#### 3.9.2. Hardness and Weight Loss Rate

As shown in Figure 5b, the hardness of all treatment groups decreased gradually, and the control group consistently exhibited lower hardness than other groups. During storage, weight loss rates increased in all treatments, with the control and *B. subtilis* suspension groups showing significantly higher rates (*p* < 0.05) than the 2.0% SA and *B. subtilis*/SA coated groups. Pomelo has soft skin that is prone to damage during handling and features large surface stomata. The coating treatments formed a protective film that blocks pathogen invasion and acts as a moisture barrier. As shown in Figure 5c, the CK group had significantly lower final hardness (9.16 ± 0.87 N) and higher weight loss than other groups, while the *B. subtilis*/SA coated group showed the highest hardness (18.62 ± 0.55 N) and lowest weight loss (7.6 ± 0.91%).

#### 3.9.3. TSS and TA

As shown in Figure 5d, the TSS content of all treatment groups and the CK exhibited an initial decrease followed by an increase. During 0–30 d, TSS increased by 1.27 ± 0.12% (2.0% SA), 1.53 ± 0.11% (*B. subtilis*/SA), 0.76 ± 0.12% (*B. subtilis* suspension), and 0.63 ± 0.16% (CK), reaching peak values. The initial TSS increase likely reflects postharvest ripening, where residual metabolic activity drives polysaccharide hydrolysis into soluble sugars. During this phase, sugar production surpasses respiratory consumption, resulting in net accumulation. The subsequent TSS decline resulted from progressive nutrient depletion [38]. By 90 d, TSS declined as follows: 2.0% SA (12.60 ± 0.24% → 10.20 ± 0.52%), *B. subtilis* suspension (11.98 ± 0.21% → 8.57 ± 0.53%), *B. subtilis*/SA coating (12.87 ± 0.23% → 10.62 ± 0.53%), and CK (11.82 ± 0.12% → 8.14 ± 0.42%). SA forms a surface barrier, while *B. subtilis* in the coating inhibits fungal activity, providing peel protection.

Organic acids serve as key respiratory substrates during storage, participating in biochemical reactions and supplying metabolic intermediates. As shown in Figure 5e, TA declined in all groups, but coated fruits maintained significantly higher levels than the control. Similarly, Linh et al. [39] reported that TA of all pomelos decreased gradually over 6 d of storage; yet, the TA in coated samples was significantly higher than the control groups. The CK showed the steepest reduction (0.96 ± 0.05 → 0.37 ± 0.08 mg/g), while the *B. subtilis*/SA coating had the smallest decline (0.93 ± 0.06 → 0.76 ± 0.05 mg/g). These results demonstrated that the coating effectively slowed the declination of TA [40].

### 3.10. Effect of B. subtilis/SA Coating on Total Phenolic and Flavonoid Contents in Wendan Pomelo Peel

**Figure 6:**
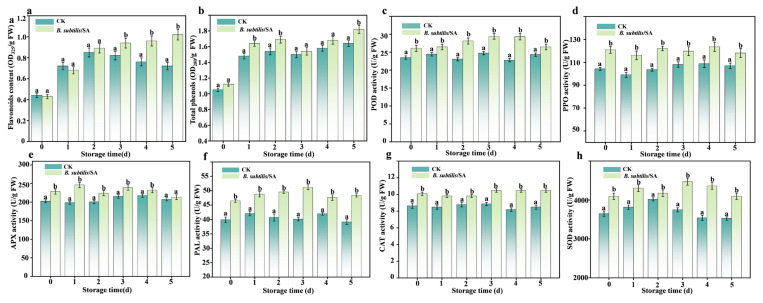
Effect of *B subtilis*/SA coating on the content of flavonoids (**a**) and total phenols (**b**) in the pomelo peel. The effect of *B. subtilis*/SA coating on resistance related enzyme activity at fruit wound site POD (**c**), PPO (**d**), APX (**e**), PAL (**f**), CAT (**g**), and SOD (**h**). The data are shown as mean ± S.E. (n = 3) and different letters (a,b) represent significant differences (*p * <  0.05).

Flavonoids are polyphenols with antioxidant activity and antiviral and bacterial effects, which can inhibit the growth of mycelium and the germination of spores [41]. As shown in Figure 6a, the flavonoid content in the peel of *B. subtilis*/SA-coated Wendan pomelo gradually increased over time. In contrast, the CK group exhibited steady flavonoid accumulation during the first 3 d, followed by a decline. By 3 d, flavonoid levels in the treated group surpassed those in the CK group. Similarly, Li et al. [38] reported that the accumulation of phenols and flavonoids in citrus fruits is associated with the induction of resistance to postharvest diseases. Phenolic substances also play a significant role in the stress resistance physiology of plants, mainly by eliminating or controlling oxygen free radicals produced by stress, thereby protecting the plant body from damage [42]. Figure 6b showed that the total phenolic content in the treated group was consistently higher than that in the CK group. In the treatment group, phenolic levels initially increased, then decreased between 0 and 3 d, and increased again slightly after 3 d. CK showed an initial increase, followed by a decline, and a subsequent steady rise after 3 d. Lin et al. [43] found that exogenous proanthocyanidin (PAs) treatment could significantly increase the content of total flavonoids and total phenols in strawberry fruits and enhance the non-enzymatic antioxidant system of strawberries. These results demonstrate that the *B. subtilis*/SA coating promotes the accumulation of flavonoids and total phenolics in the pomelo peel, which may contribute to enhance disease resistance.

### 3.11. Effects of B. subtilis/SA Coating on Resistance-Related Metabolites and Enzyme Activities in Wendan Pomelo

As shown in Figure 6c–h, the activities of POD, PPO, APX, PAL, CAT, and SOD at wound sites of *B. subtilis*/SA-coated pomelos were consistently higher than the CK group. In the treatment group, POD activity peaked on 3 d (29.41 ± 0.45 U/g FW), while PPO and APX activities reached maxima on 4 d (133.54 ± 4.16 U/g FW and 239.24 ± 7.17 U/g FW). PAL, CAT, and SOD activities peaked on 3 d (51.19 ± 0.82, 10.48 ± 0.21, and 4470.91 ± 89.42 U/g FW), followed by a decline. In the CK group, POD peaked on 3 d (24.73 ± 0.45 U/g FW), PPO and APX on 4 d (118.73 ± 3.37 and 217.28 ± 4.34 U/g FW), PAL on 4 d (41.96 ± 0.46 U/g FW), CAT on 3 d (8.87 ± 0.26 U/g FW), and SOD on 2 d (4016.85 ± 40.51 U/g FW), after which activities declined. By 3 d, POD, CAT, and SOD peaked in both groups, with the treatment group exhibiting 1.19-, 1.18-, and 1.11-fold higher activities. APX activity in the CK group peaked on 4 d, whereas the treatment group peaked earlier (2 d), showing 1.13-fold higher activity. Conversely, PAL in the control peaked on 2 d, while the treatment group peaked later (4 d) with 1.21-fold higher activity. Both groups exhibited peak PPO activity on 4 d, with the treatment group showing 1.12-fold higher activity. Notably, enzyme activities in the treatment group were significantly higher than the CK group. These results demonstrated that the coating enhances fruit resistance by promoting resistance-compound accumulation and activating defense enzymes [39].

## 4. Conclusions

The synergistic application of *B. subtilis* and SA coatings demonstrated significant potential for enhancing the postharvest preservation of Wendan pomelo. The primary pathogenic fungi causing postharvest decay were identified as *P. digitatum* and *P. italicum*, with *B. subtilis* exhibiting potent antagonistic activity via the secretion of extracellular enzymes. The SA-based coating (2.0% concentration) provided optimal physicochemical properties and biocompatibility, maintaining *B. subtilis* viability without compromising mechanical or barrier performance. *B. subtilis*/SA coating effectively delayed quality deterioration. The *B. subtilis*/SA coating treatment not only reduced the incidence of postharvest diseases through its barrier properties, but also enhanced preservation effects in pomelo by promoting the accumulation of resistance-related compounds such as flavonoids and total phenols, as well as increasing the enzymatic activities of APX, SOD, POD, and CAT. This study provides a theoretical foundation for bio-based preservation strategies for citrus fruits, emphasizing the dual roles of physical barriers and biological induction mechanisms. While this study provides insights into the efficacy of the *B. subtilis*/SA coating, further investigations could expand its scope and applicability. Future work should evaluate a broader range of *B. subtilis* strains to identify potential enhancements in antifungal performance. Additionally, assessing preservative effects of the coating under room temperature conditions would better reflect real-world storage scenarios. Finally, extending validation to other citrus varieties will be essential to determine the generalizability of the treatment across species.

## Figures and Tables

**Table 1:** Determination of in vivo antifungal activity.

	Radius of Area of Incidence (cm)					
	0 d	1 d	2 d	3 d	4 d	5 d
CK	-	-	1.62 ± 0.12 ^a^	4.74 ± 0.08 ^a^	6.43 ± 0.23 ^a^	9.46 ± 0.21 ^a^
2.0% SA	-	-	-	-	1.26 ± 0.04 ^b^	1.82 ± 0.13 ^b^
*B. subtilis*/SA	-	-	-	-	-	-

Note: All results were expressed as mean ± standard deviation; the letters a,b in the same column indicated significant differences in the data (*p* < 0.05); “-” indicates that no disease occurred at the wound site of the fruit.

## Data Availability

The original contributions presented in this study are included in the article/Supplementary Materials. Further inquiries can be directed to the corresponding authors.

## Supplementary Materials

The following supporting information can be downloaded at: https://www.mdpi.com/article/10.3390/foods14193303/s1. Figure S1. Morphological characteristics of antagonistic bacteria (a). Phylogenetic tree of antagonistic bacterium JK-1 (b); Figure S2. CK group (a_1_), CS (a_2_), SA (a_3_), KGM (a_4_), CMC-Na (a_5_). The influence of different coating substrates on the morphology of *B. subtilis*. Antibacterial activity of *B. subtilis* suspension with different concentrations against pathogenic bacteria in vitro (b). Appearance of fruits treated in vivo with different concentrations of *B. subtilis* bacterial suspension (c). The data is shown as mean ± S.E. (n = 3) and different letters (a–f) represent the significant differences (*p*  <  0.05); Figure S3. In vivo antibacterial activity of CK group (A), SA (B) and *B. subtilis*/SA coatings (C); Figure S4. Changes in the L* (a), a* (b), b* (c) of Wendan pomelo during storage; Table S1 Changes in the color of blueberries during storage; Table S2 Changes in the hardness, weight loss rate TSS and TA of blueberries during storage.
