# Supplementary material for: Preserving Pomelo Quality: Sodium Alginate Coating Containing Bacillus subtilis for Sustained Antifungal Activity
Source: Foods. 2025 Sep 24;14(19):3303. doi: 10.3390/foods14193303 (PMC12523436; doi:10.3390/foods14193303)
Supplement: Supplementary file 1 [file foods-14-03303-s001.zip › foods-3869296-supplementary.pdf]

Mechanism of sodium alginate coating containing *Bacillus subtilis* for sustained antifungal activity to preserve pomelo quality

Xi Wei [1], Yan Liu [1], Tingting Tang [2], Shanshan Lei [1], Jing Wu [1], Tianhua Ding [1], Xiaoyi Zhu [1], Weirui Fang [1], Jiayi Zheng [1], Yuxin Liu [1], Wen Qin [1], Mingrui Chen [1,*] and Yaowen Liu [1,*]

[1] College of Food Science, Sichuan Agricultural University, Yaan, China
[2] College of Agriculture and Forestry Science and Technology, Chongqing Three Gorges Vocational College, Chongqing, China
* Correspondence: mingrui.chen@sicau.edu.cn (M. Chen); yaowenliu@sicau.edu.cn (Y. Liu)

Supplemental materials

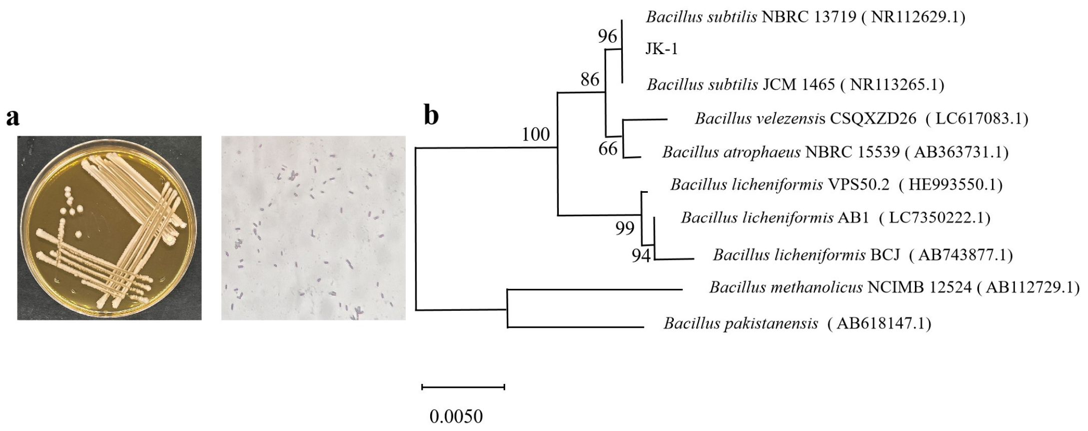

Figure S1. Morphological characteristics of antagonistic bacteria (a). Phylogenetic tree of antagonistic bacterium JK-1 (b).

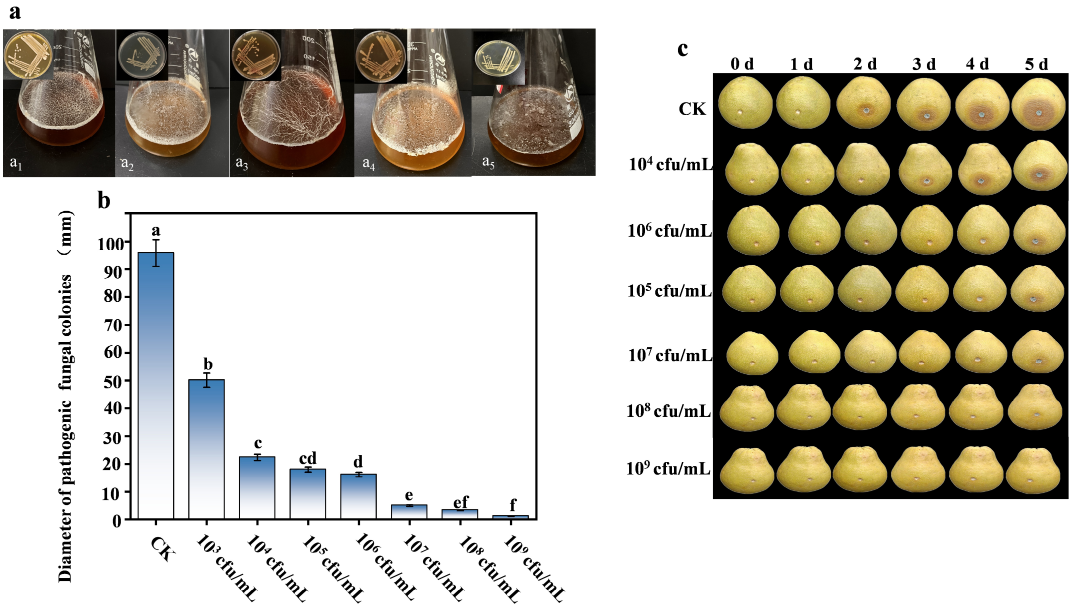

Figure S2. CK group (a_1), CS (a_2), SA (a_3), KGM (a_4), CMC-Na (a_5). The influence of different coating substrates on the morphology of *B. subtilis*. Antibacterial activity of *B. subtilis* suspension with different concentrations against pathogenic bacteria in vitro (b). Appearance of fruits treated in vivo with different concentrations of *B. subtilis* bacterial suspension (c). The data is shown as mean ± S.E. (n = 3) and different letters (a-f) represent the significant differ-ences ($p < 0.05$).

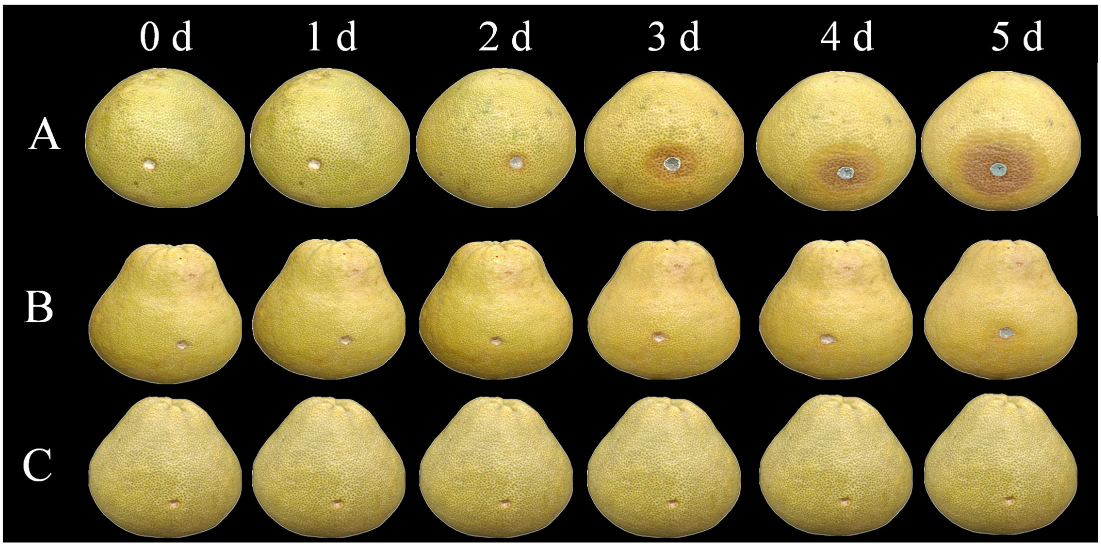

Figure S3. *In vivo* antibacterial activity of CK group (A), SA (B) and *B. subtilis*/SA coatings (C).

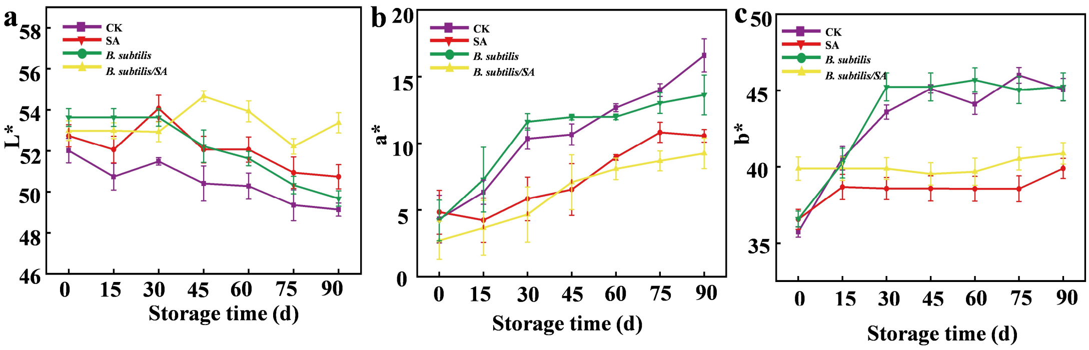

Figure S4. Changes in the L* (a), a* (b), b* (c) of Wendan pomelo during storage. Detailed data and statistics are provided in supplementary Table S2.

Table S1 Changes in the color of blueberries during storage.

	Groups	0 d	15 d	30 d	45 d	60 d	75 d	90d
L*	CK	52.02±0.60^{b}	50.74±0.65^{c}	51.49±0.18^{c}	50.41±0.86^{c}	50.28±0.64^{c}	49.35±0.75^{c}	49.13±0.32^{c}
	SA	52.74±0.54^{ab}	52.07±0.65^{b}	54.07±0.64^{a}	52.07±0.65^{b}	52.07±0.62^{b}	50.94±0.77^{b}	50.74±0.59^{b}
	B. subtilis	53.63±0.43^{a}	53.63±0.43^{a}	53.63±0.42^{ab}	52.23±0.79^{b}	51.63±0.35^{b}	50.33±0.43^{bc}	49.67±0.38^{c}
	B. subtilis/SA	52.99±0.48^{ab}	52.99±0.38^{ab}	52.93±0.48^{b}	54.66±0.26^{a}	53.93±0.51^{a}	52.23±0.38^{a}	53.37±0.49^{a}
a*	CK	4.33±1.78^{a}	6.33±0.91^{a}	10.36±0.79^{a}	10.67±0.79^{a}	12.67±0.29^{a}	13.99±0.48^{a}	16.57±1.23^{a}
	SA	4.84±1.64^{a}	4.24±1.64^{b}	5.84±1.63^{b}	6.54±1.94^{b}	8.95±0.21^{b}	10.83±0.75^{b}	10.57±0.47^{c}
	B. subtilis	4.23±1.51^{a}	7.29±2.44^{a}	11.62±0.61^{a}	11.97±0.21^{a}	11.99±0.21^{a}	13.02±0.77^{a}	13.63±1.49^{b}
	B. subtilis/SA	2.73±1.43^{a}	3.67±2.06^{b}	4.67±2.06^{b}	7.09±2.06^{b}	8.09±0.8^{c}	8.69±0.75^{c}	9.26±1.15^{c}
b*	CK	35.73±0.34^{b}	40.56±0.77^{a}	43.59±0.46^{b}	45.11±0.27^{a}	44.11±0.67^{b}	45.99±0.51^{a}	45.05±0.73^{a}
	SA	36.55±0.65^{b}	38.67±0.83^{b}	38.57±0.73^{d}	38.57±0.83^{b}	38.55±0.82^{c}	38.55±0.85^{c}	39.89±0.66^{b}
	B. subtilis	36.57±0.52^{b}	40.24±0.98^{ab}	45.23±0.91^{a}	45.23±0.91^{a}	45.68±0.79^{a}	45.03±0.89^{a}	45.24±0.91^{a}
	B. subtilis/SA	39.88±0.77^{a}	39.88±0.77^{ab}	39.88±0.71^{c}	39.53±0.74^{b}	39.67±0.89^{c}	40.53±0.74^{b}	40.88±0.71^{b}

Different letters indicate significant differences between treatment groups at same point ($p < 0.05$, n=3)

Table S2 Changes in the hardness, weight loss rate TSS and TA of blueberries during storage.

	Groups	0 d	15 d	30 d	45 d	60 d	75 d	90d
Hardness (N)	CK	28.45±0.93^{a}	19.45±1.85^{c}	15.67±0.68^{c}	14.60±0.12^{c}	10.39±0.44^{c}	10.06±0.48^{d}	9.16±0.88^{c}
	SA	27.67±0.70^{ab}	26.58±0.19^{a}	22.81±0.91^{a}	20.89±0.73^{b}	19.92±0.15^{a}	17.81±0.63^{b}	14.17±0.29^{b}
	B. subtilis	26.85±0.33^{b}	24.18±0.27^{b}	17.85±0.58^{b}	13.60±0.45^{c}	11.98±0.73^{b}	11.83±0.16^{c}	10.14±0.98^{c}
	B. subtilis/SA	27.79±0.67^{ab}	24.45±0.17^{b}	23.60±1.15^{a}	22.91±1.97^{a}	19.18±0.49^{a}	18.88±0.29^{a}	18.62±0.55^{a}
Weight loss rate (%)	CK	0.00±1.13^{a}	2.80±0.53^{a}	6.00±1.96^{a}	9.80±1.12^{a}	14.00±1.02^{a}	17.00±1.92^{a}	23.00±2.94^{a}
	SA	0.00±1.13^{a}	1.60±0.56^{a}	2.40±0.32^{b}	5.70±1.47^{b}	6.50±2.23^{b}	7.10±1.81^{b}	7.90±1.91^{b}
	B. subtilis	0.00±1.13^{a}	1.70±0.95^{a}	2.30±1.86^{b}	4.90±1.87^{b}	6.40±1.82^{b}	6.80±1.74^{b}	7.60±1.71^{b}
	B. subtilis/SA	0.00±1.13^{a}	1.70±0.95^{a}	2.30±1.86^{b}	4.90±1.87^{b}	6.40±1.82^{b}	6.80±1.74^{b}	7.60±1.71^{b}
TSS (%)	CK	11.07±0.57^{a}	11.22±0.34^{b}	11.71±0.45^{c}	10.67±0.31^{b}	10.12±0.24^{b}	9.27±0.31^{b}	8.13±0.33^{b}
	SA	11.33±0.57^{a}	11.93±0.47^{a}	12.65±0.21^{ab}	11.84±0.52^{a}	11.67±0.43^{a}	10.93±0.11^{a}	10.24±0.55^{a}
	B. subtilis	11.33±0.57^{a}	11.87±0.17^{ab}	12.32±0.13^{b}	10.93±0.11^{b}	9.93±0.17^{b}	9.61±0.43^{b}	8.57±0.85^{b}
	B. subtilis/SA	11.33±0.57^{a}	12.13±0.24^{a}	12.87±0.17^{a}	12.21±0.34^{a}	11.67±0.55^{a}	11.12±0.45^{a}	10.62±0.55^{a}
TA (mg/g)	CK	0.97±0.02^{a}	0.67±0.02^{b}	0.62±0.02^{c}	0.56±0.02^{b}	0.49±0.01^{c}	0.40±0.02^{d}	0.37±0.02^{d}
	SA	0.91±0.02^{b}	0.86±0.02^{a}	0.81±0.02^{a}	0.76±0.03^{a}	0.76±0.03^{a}	0.67±0.02^{b}	0.67±0.01^{b}
	B. subtilis	0.91±0.01^{b}	0.87±0.06^{a}	0.69±0.02^{b}	0.59±0.01^{b}	0.56±0.01^{b}	0.49±0.02^{c}	0.42±0.01^{c}
	B. subtilis/SA	0.94±0.02^{ab}	0.86±0.02^{a}	0.80±0.01^{a}	0.77±0.04^{a}	0.77±0.03^{a}	0.74±0.02^{a}	0.77±0.01^{a}

Different letters indicate significant differences between treatment groups at same point ($p < 0.05$, n=3)
